# Bioassay-Guided Fractionation of a Leaf Extract from *Combretum mucronatum* with Anthelmintic Activity: Oligomeric Procyanidins as the Active Principle

**DOI:** 10.3390/molecules200814810

**Published:** 2015-08-14

**Authors:** Verena Spiegler, Jandirk Sendker, Frank Petereit, Eva Liebau, Andreas Hensel

**Affiliations:** 1 Institute for Pharmaceutical Biology and Phytochemistry, University of Münster, Schlossplatz 2, D-48149 Münster, Germany; E-Mails: verena.spiegler@uni-muenster.de (V.S.); jandirk.sendker@uni-muenster.de (J.S.); frank.petereit@uni-muenster.de (F.P.); 2 Institute for Zoophysiology, University of Münster, Schlossplatz 8, D-48143 Münster, Germany; E-Mail: liebaue@uni-muenster.de

**Keywords:** *Combretum mucronatum*, anthelmintic, *Caenorhabditis elegans*, flavonoids, proanthocyanidins, structure-activity, tannins

## Abstract

*Combretum mucronatum* Schumach. & Thonn. is a medicinal plant widely used in West African traditional medicine for wound healing and the treatment of helminth infections. The present study aimed at a phytochemical characterization of a hydroalcoholic leaf extract of this plant and the identification of the anthelmintic compounds by bioassay-guided fractionation. An EtOH-H_2_O (1:1) extract from defatted leaves was partitioned between EtOAc and H_2_O. Further fractionation was performed by fast centrifugal partition chromatography, RP18-MPLC and HPLC. Epicatechin (**1**), oligomeric proanthocyanidins (OPC) **2** to **10** (mainly procyanidins) and flavonoids **11** to **13** were identified as main components of the extract. The hydroalcoholic extract, fractions and purified compounds were tested *in vitro* for their anthelmintic activity using the model nematode *Caenorhabditis elegans*. The bioassay-guided fractionation led to the identification of OPCs as the active compounds with a dose-dependent anthelmintic activity ranging from 1 to 1000 μM. Using OPC-clusters with a defined degree of polymerization (DP) revealed that a DP ≥ 3 is necessary for an anthelmintic activity, whereas a DP > 4 does not lead to a further increased inhibitory effect against the helminths. In summary, the findings rationalize the traditional use of *C. mucronatum* and provide further insight into the anthelmintic activity of condensed tannins.

## 1. Introduction

Approximately 1.5 billion people worldwide suffer from infestations with soil-transmitted helminths (STH) [1], with *Ascaris lumbricoides*, *Trichuris trichiura* and *Ancylostoma duodenale* being the most common parasites [2]. Most people affected live in less developed countries of Sub-Saharan Africa, South America and South East Asia, where poverty, along with poor sanitary conditions, give rise to infections with intestinal helminths. Although not lethal in most cases, these parasites can cause considerable morbidity, such as anaemia and malnutrition, leading to decreased growth and cognitive retardation, especially in children in endemic countries [3,4].

The WHO is currently tackling these problems by setting up Mass Drug Administration (MDA) programs that aim at preventively treating school-aged and preschool-aged children with broad spectrum anthelmintics. Although providing access to effective treatments is desirable for all people affected by these parasites, the long term efficacy remains undetermined and large-scale preventive actions also bear the risk of resistances against the respective drugs to emerge [5,6,7]. This in turn will strongly limit the effective use of the very limited number of drugs against STH we are mainly relying on, namely albendazole, mebendazole, levamisole and pyrantel pamoate [8]. While at present the situation regarding resistances is not as severe as in veterinary medicine, monitoring of the drug efficacy should be improved and efforts in the development of new drugs be stepped up [9].

Natural products have ever since been a valuable source for the identification and the development of new lead structures against various targets, including helminths [10,11]. One approach to discover new active compounds is the investigation of plants based on their traditional usage by an *in vitro* confirmation of their respective bioactivity followed by advanced functional and phytochemical studies leading to an isolation of the potential active principles [10].

Therefore, an ethnopharmacological field study was carried out from October 2012 to February 2013 in the Ashanti region in central Ghana which revealed a leaf extract of *Combretum mucronatum* Schumach. & Thonn. to be among the most frequently used plant preparation against helminths [12]. The *in vitro* activity of a crude ethanolic extract was shown to be superior to other plant preparations against different kinds of nematodes, including *Caenorhabditis elegans* [12,13], but despite an entry of this plant in the Ghana Herbal Pharmacopoeia, knowledge about its phytochemistry and functionality is very limited. Recently, phytochemical investigations by Kisseih *et al.* revealed the presence of procyanidins and flavonoids, fatty acids, organic acids and carbohydrates as major components of the leaves of *C. mucronatum* [14]. Additionally, extracts from several other *Combretum* species have been assessed for their anthelmintic properties [15], but to our knowledge, no linkage has been established between defined compounds from the investigated extracts of the *Combretum* species and a potential anthelminthic bioactivity.

This study aims at gaining further insight into the phytochemical composition of a hydro-ethanolic leaf extract of *C. mucronatum* and at the identification of the active principles responsible for the anthelmintic activity by a bioassay-guided fractionation.

## 2. Results and Discussion

### 2.1. Phytochemical Characterization of a Hydroethanolic Leaf Extract from C. mucronatum

Although the identification of the active compounds was one of the goals in this study, we did not perform a bioassay-guided fractionation in the strict sense. This method is one of the most common techniques to identify bioactive compounds from complex mixtures such as extracts by one or more separation steps accompanied by activity tests to select active fractions for further subfractionation (for review see [16]). In our case this would mean that while focusing entirely on the bioactivity, inactive components of the plant extract which have not been characterized yet would remain unexplored. For that reason, we performed the fractionation by testing the anthelmintic activity after each separation step, but additionally included the isolation and identification of inactive or less active compounds for an improved phytochemical characterization of the extract.

**Figure 1:**
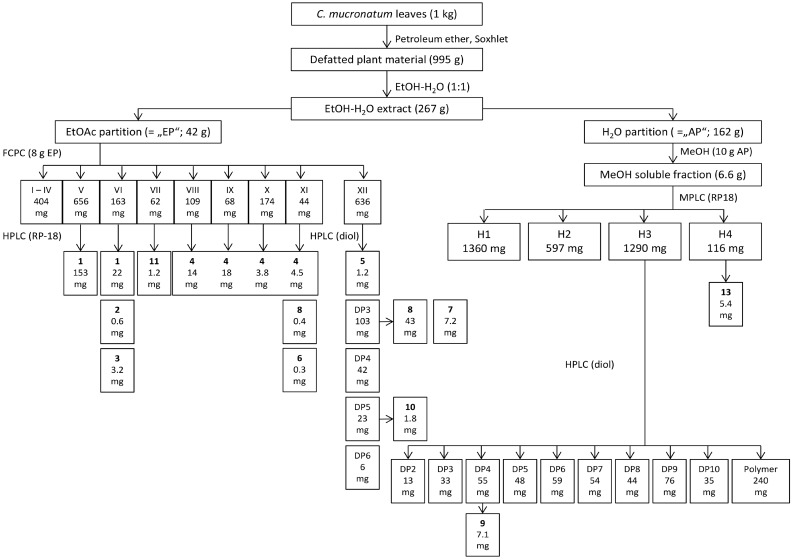
Extraction and fractionation scheme of leaves from *Combretum mucronatum* as described in the materials parts 3.3 to 3.5 of this manuscript.

As summarized in Figure 1, dried leaves were defatted and extracted by ethanol-water (1:1), followed by partitioning of the extract between ethyl acetate (EtOAc) and water. This protocol yielded an EtOAc fraction, mainly composed of flavonoids and oligomeric proanthocyanidins (OPC) with a degree of polymerization (DP) ≤ six, and a more hydrophilic H_2_O fraction containing higher oligomeric and polymeric proanthocyanidins, flavonoids and carbohydrates. The EtOAc partition was further fractionated by FCPC to yield 11 fractions (I to XI) from the mobile phase and one additional fraction (XII) formed by the remaining stationary phase. TLC analysis indicated the presence of flavan-3-ols, dimeric and trimeric proanthocyanidins and flavonoids. Subsequent fractionation and isolation of purified compounds was performed by preparative HPLC on an RP18 stationary phase, followed by identification of the purified compounds by NMR and spectroscopic means (CD, ESI-MS). All dimeric proanthocyanidins were identified by ^1^H-NMR of the respective peracetates in comparison to published data [17,18].

**Figure 2:**
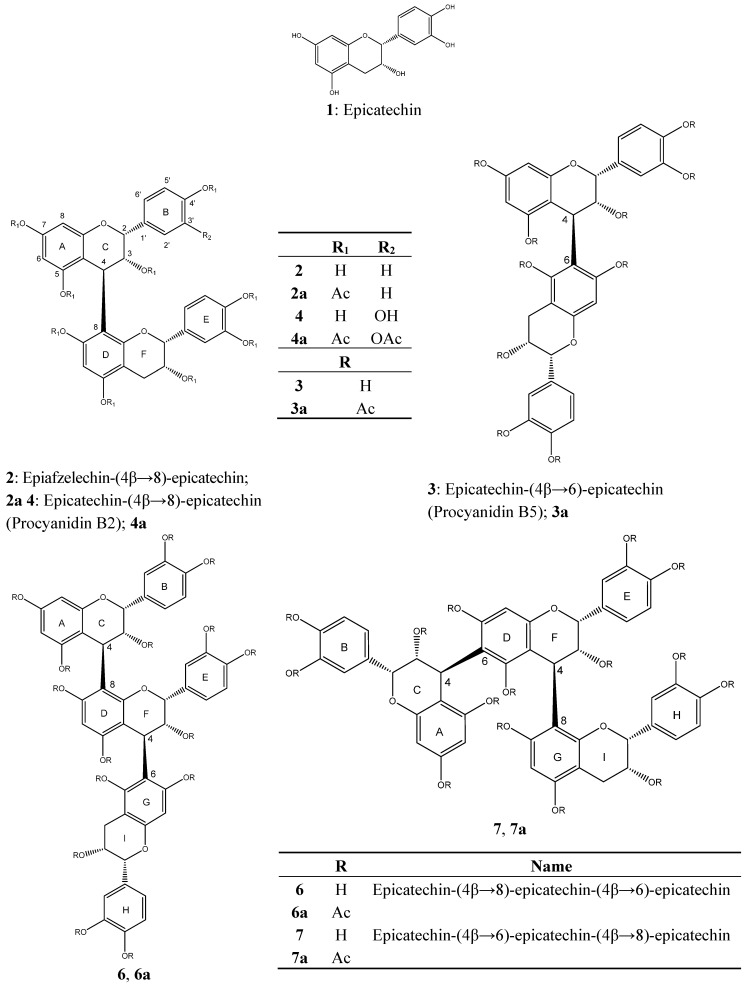

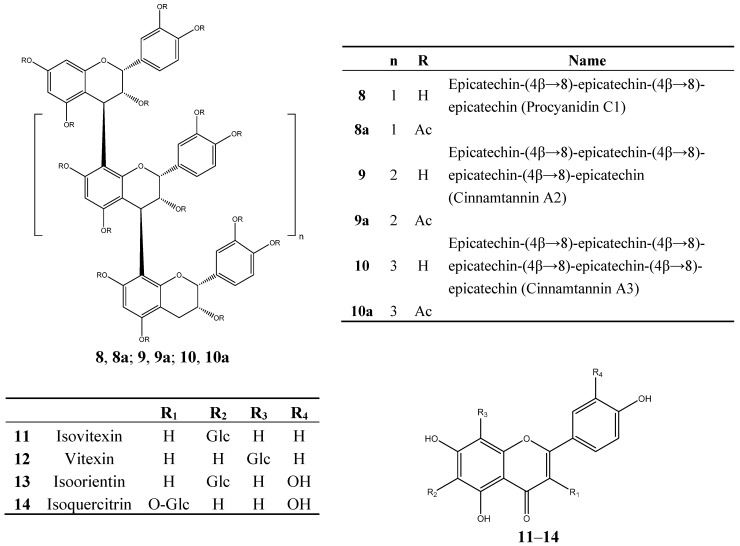
Structural features of the oligomeric proanthocyanidins, proanthocyanidin peracteates and flavonoids isolated from *C. mucronatum*; peracetylated OPCs were used for unambiguous structure elucidation.

Fraction V was mainly composed of epicatechin (**1**) (Figure 2). Three compounds were purified from fraction VI: beside epicatechin (**1**) and epicatechin-(4β→6)-epicatechin (procyanidin B5, **3**), epiafzelechin-(4β→8)-epicatechin (**2**) was isolated. A further dimeric procyanidin epicatechin-(4β→8)-epicatechin (procyanidin B2, **4**) was isolated in good yields from fractions VII to XI and turned out to be the dominant OPC from *C. mucronatum* leaves (Figure 2).

Preparative RP18-HPLC of fraction XI yielded the trimeric procyanidin epicatechin-(4β→8)-epicatechin-(4β→8)-epicatechin (procyanidin C1, **8**), and interestingly**,** another trimeric procyanidin epicatechin-(4β→8)-epicatechin-(4β→6)-epicatechin (**6**).

Spectroscopic (NMR, ESI-MS, CD) identification of this trimer and further OPCs **6** to **10** obtained during subsequent isolation steps was performed after derivatization to the respective peracetates and comparison to published data. Due to a better resolution of the spectra it was also possible to assign the signals for the protons of the catechol ring for each of the three units in **6a** and **7a**. Additionally, signals of the carbon spectrum could be assigned, completing the spectroscopic data set for the peracetylated compounds [18,19].

TLC analysis of fraction XII, obtained from the FCPC stationary phase indicated the presence of a variety of OPCs. From this fraction OPC clusters with defined DP from 3 to 6 were isolated on a preparative scale in good yields by HPLC on a diol stationary phase [20,21,22,23].

From the OPC cluster DP3 epicatechin-(4β→8)-epicatechin-(4β→8)-epicatechin (procyanidin C1, **8**) (isolated also as described before in fraction XI) and epicatechin-(4β→6)-epicatechin-(4β→8)-epicatechin (**7**) were isolated. Additionally a pentameric procyanidin **10** was obtained from the OPC cluster DP5.

**Figure 3:**
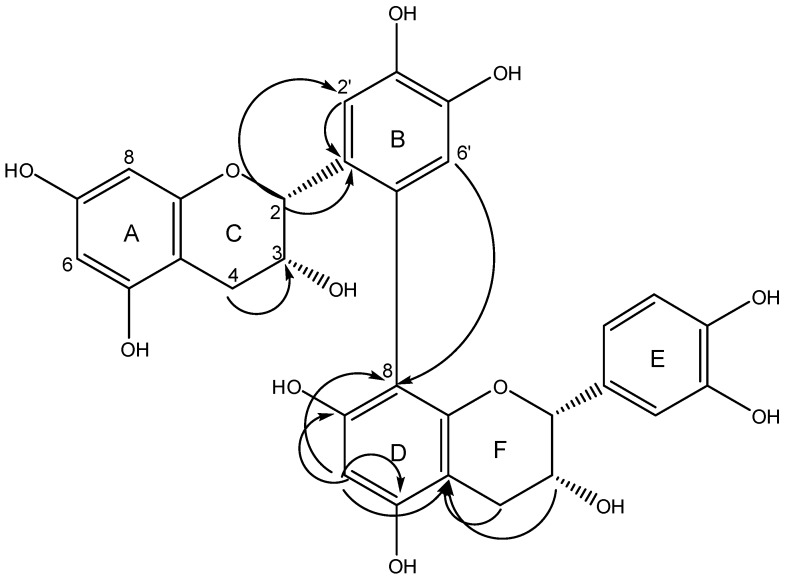
Structure of epicatechin-(6′→8)-epicatechin (**5**) isolated from *C. mucronatum*. Arrows indicate relevant HMBC correlations.

An unusual dimeric procyanidin epicatechin-(6′→8)-epicatechin (**5**, Figure 3) with a linkage between position 6′ of the B-ring of the upper epicatechin unit and position 8 of the lower epicatechin unit was isolated from fraction XII. This compound has been described as a product formed from catechin or epicatechin by autoxidation, chemical or enzymatic oxidation via formation of an *ortho*-quinone and reaction with a hydroquinone (e.g., epicatechin) in a 1,4-Michael-addition [24,25]. The C-C linkage is preferably formed between position 6′ of the quinone, which can be easily attacked by a nucleophile, and position 8 of the hydroquinone which is sterically better accessible than position 6 [25]. Nevertheless, we could not determine the exact position of the linkage in ring D directly from the spectroscopic data obtained, but concluded from comparison to literature that the two rings are linked via position 6′ and 8. This compound or similar derivatives consisting of two catechin units have been synthesized enzymatically [26,27] and non-enzymatically [28,29] and have been also isolated from grape pomace [30] and oak bark [31].

Because of these findings it seemed possible that similar B-ring linked compounds with a higher DP might be present in the *C. mucronatum* extract. However, intensive HPLC and HPLC-MS investigations gave no hints for the occurrence of such oligomers, which means that the dimer seems to be the only biflavonoid of this type. It still remains unclear, whether such biflavonoids occur in genuine plant material or whether they are formed during the drying process of the plant material, during the extraction procedure or the storage.

**Figure 4:**
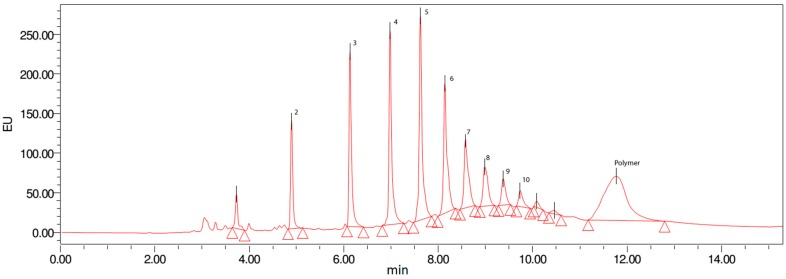
UHPLC of the OPC-enriched fraction H3. Numbers indicate the degree of polymerization of the respective OPC clusters.

From the H_2_O partition a MeOH-soluble fraction was obtained which was further separated by MPLC in 4 subfractions H1 to H4 (Figure 1). Fraction H4 was further purified by preparative HPLC and yielded pure isoorientin (**13**). H3 turned out to contain high amounts of OPCs. Analytical HPLC of H3 on diol stationary phase revealed a wide and homologues distribution of OPCs with different DPs (Figure 4).

Subsequently, H3 was fractionated by preparative HPLC using a diol stationary phase for separation of distinct OPC clusters with defined DP [20,21,22,23]. This protocol yielded procyanidin clusters from DP2 to DP10 and a polymer fraction in good yields (Figure 1). All clusters isolated were investigated by LC-MS concerning their respective masses which indicated the presence of B-type procyanidins; the existence of A-type linkages was excluded. 

The tetrameric procyanidin cinnamtannin A2 (**9**; despite the term “A”, cinnamtannins “A2” and “A3” are B-type procyanidins) and the pentameric cinnamtannin A3 (**10**) were identified as the major compounds obtained from the OPC clusters DP4 (obtained from the H_2_O partition) and DP5 (obtained from the EtOAc partition) and were identified in form of the respective peracetates (**9a** and **10a**). Data for **9a** corresponds well to literature [25], whereas **10a** could not be identified unambiguously, due to the limited amount of substance available for NMR and the lack of reference data for the peracetylated derivative. Based on the findings of the isolated OPC DP2 to DP4, the major component of each cluster consists of (4β→8)-linked epicatechin building blocks, whereas OPCs with a (4β→6) linkage were obtained in much lower yields. Therefore, we assumed that the main peak in the chromatogram of the OPC cluster DP5 should correspond to an epicatechin pentamer with a (4β→8) linkage. In the next step we tried to confirm this assumption by 1D (^1^H, ^13^C) and 2D (COSY, NOE, HMBC and HSCQ) NMR experiments, still, it was not possible to completely assign signals for all of the protons and carbons of the molecule due to the low amount of substance available for structure elucidation.

All OPC procyanidin clusters with defined DP were used in the following functional investigations for potential anthelminitic activity.

The Ghana Herbal Pharmacopoeia mentions a (2*R*,3*S*)-configured “combretum-catechin” as one of the chemical constituents of this medicinal plant [32]. Our findings however do not support the occurrence of (2*R*,3*S*)-flavan-3-ols, as all components identified are exclusively composed of (2*R*,3*R*)-flavan-3-ol units. 

Triterpene saponins of the dammarane type [33], oleanane type [34] or cycloartane type [35] have been previously described for various other *Combretum* species, yet, investigations of the EtOH-H_2_O extract and various fractions by mass spectrometry did not reveal the presence of saponins in the leaf extract of *C. mucronatum*.

Concerning the flavonoid content of the extracts, isovitexin (**11**) was isolated from fraction V as the most abundant flavonoid in the extract. Additionally, isoorientin (**13**) was obtained from fraction H4 of the H_2_O-partition and identified by spectroscopic analysis (NMR and MS). TLC and HPLC analysis of fractions VII to XI also revealed the presence of different flavonoids, which unfortunately could not be isolated on a preparative scale due to their similar retention times in preparative HPLC. Therefore, these flavonoids were identified by analytical HPLC as vitexin (**12**), isoorientin (**13**) and isoquercitrin (**14**) by spiking of the test solutions with a set of flavonoid reference compounds. 

### 2.2. Bioassay-Guided Fractionation

L4 larvae and young adults of the free-living nematode *C. elegans* were used to assess the survival rate of the worms *in vitro*. Although not parasitic, it is closely related to certain parasites and is used worldwide as a well-established model organism for anthelmintic tests. Its short life cycle and easy maintenance under lab conditions are the main advantages compared to parasitic nematodes that usually require animal hosts for maintenance and propagation [36,37].The hydroethanolic extract of the leaves of *C. mucronatum* showed moderate anthelmintic activity, with an LC_50_ of 1.67 mg/mL. Subsequent testing of the EtOAc and H_2_O partition indicated that the active components were mainly located in the more lipophilic ethyl acetate fraction with an LC_50_ = 1.73 mg/mL compared to the aqueous fraction for which an LC_50_ could not be determined due to its weak activity. Generally, the LC_50_ values obtained in this study might seem quite high compared to results from other test systems, but despite its advantages in lab work *C. elegans* is known to be more resistant to drug treatment than other nematodes. For example, standard anthelmintic drugs such as albendazole and ivermectin were either shown to be inactive *in vitro* or require incubation over several days at concentrations in the mM range [38]. This also applies to the positive control levamisole-HCl (40 mM, approx. 14.5 mg/mL) used in this study for which the concentration is approximately 10-fold that of its therapeutic use.

Further fractionation of the EtOAc partition by FCPC was performed and fractions V to XII showed anthelmintic effects with the activity increasing from V to XII. Phytochemical investigations by TLC and UHPLC revealed condensed tannins and flavonoids to be the major constituents of these fractions. As all active fractions were dominated by flavan-3ols (V) and oligomeric procyanidins (VI to XII) it was assumed that the OPCs contribute significantly to the anthelmintic activity. To prove this hypothesis OPCs were quantitatively removed from the EtOAc partition using polyvinylpyrrolidone (PVPP), followed by functional testing of the remaining OPC-free fraction. As expected, this OPC-depleted fraction (absence of OPCs had been proven by TLC and HPLC studies) had no anthelmintic activity at all (concentrations tested up to 5 mg/mL). This clearly indicates that condensed tannins are responsible for the anthelmintic activity of *C. mucronatum* leaves.

Nevertheless, results from other investigations showed an activity of flavonols and flavonolglycosides against *Haemonchus contortus* [39], therefore we cannot rule out any synergistic effects by the flavonoids found in *C. mucronatum*, although they were shown not to be directly active.

As observed in previous studies, the H_2_O partition obtained from the hydroalcoholic extract was expected to contain proanthocyanidins of higher molecular weight [17,23] and the molecular size of OPCs has been reported to be one major factor responsible for the bioactivity of condensed tannins in general [40] as well as for their anthelmintic activity [41,42]. Therefore, the MeOH-soluble subfraction of the H_2_O partition was further fractionated by MPLC despite its limited activity against *C. elegans* to yield OPC clusters with distinct DPs from 3 to 10 and a polymeric fraction (Figure 4).

With the exception of one dimeric proanthocyanidin with an epiafzelechin unit (compound **2**) all other OPCs are entirely composed of epicatechin as building blocks (Figure 1). Compared to extracts from other tannin-rich plants which often show a broader variety in their molecular composition, e.g., catechin units beside epicatechin, mono-, di- and trihydroxylation of the B-ring or A-type linkages, the only variation among the OPCs isolated from *C. mucronatum* seems to be the type of linkage between the epicatechin units. This uniform pattern is an advantage for the determination of structure-activity relations, since it is possible to focus entirely on the role of the molecular size of the procyanidins. Until now, effects of condensed tannins against different kinds of nematodes have been subject of several investigations, but either the compounds tested did not exceed a DP ≥ 5 [41,43] or bioassays were performed using purified and well characterized fractions of condensed tannins [42,44,45], but not isolated compounds.

**Figure 5:**
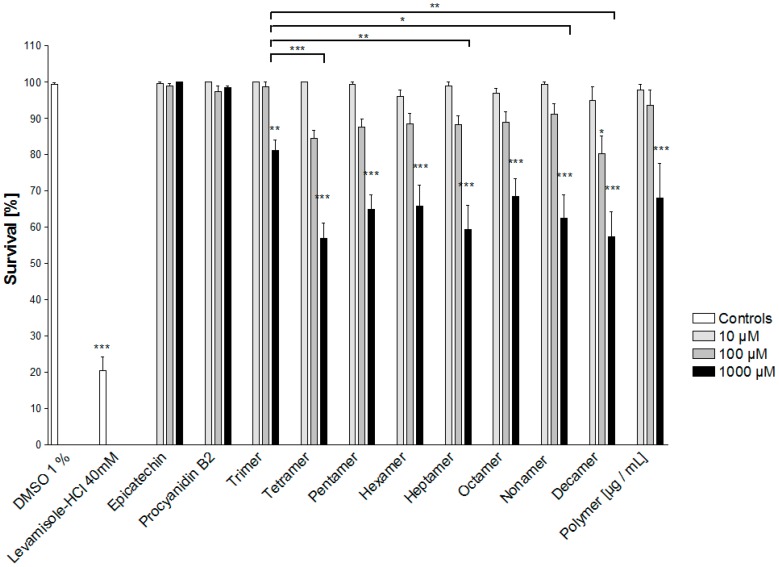
Anthelmintic activity of isolated procyanidin clusters against *C. elegans.* Untreated *C. elegans* in test medium containing 1% DMSO served as negative control (NC), levamisole-HCl (40 mM) served as positive control. ** *p* < 0.01, *** *p* < 0.001 compared to NC. Brackets indicate significant differences in the activity among the OPC clusters DP3 to DP10 (* *p* < 0.05, ** *p* < 0.01, *** *p* < 0.001).

Compounds **1**, **4**, clusters of DP 3 to 10 and the polymeric fraction were assayed under *in vitro* conditions to determine the influence of the respective molecular size on the anthelmintic activity (Figure 5).

**Figure 6:**
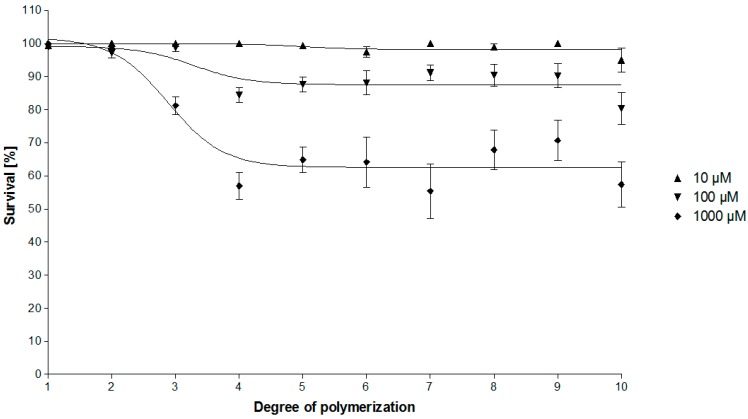
Anthelmintic activity as a function of the DP of the OPC from *C. mucronatum*. The survival rate of the worms decreases up to DP4, but no further decrease can be observed up to DP10.

While epicatechin and the dimeric procyanidin B2 turned out to be inactive at all concentrations tested (10 to 1000 μM), the survival rate of the worms decreased significantly when placed in contact with OPCs of a DP ≥ 3. On the other hand, no significant differences among OPC clusters with DP4 to DP10 were observed, indicating that a certain degree of oligomerization is necessary for the anthelmintic activity of OPCs, but once the number of epicatechin units exceeds four, no further increase in bioactivity against *C. elegans* occurs. Interestingly, the OPC polymer was not significantly different to the OPC clusters DP4 to 10, although we had expected its activity to be superior. A similar finding has previously been explained by the strongly reduced solubility of such polymers in aqueous systems [46]. Figure 6 correlates the DP of the different OPCs against their respective anthelmintic activity, confirming the observation that the best anthelmintic activity is mediated by procyanidin clusters DP > 3.

These findings are in accordance with general observations regarding the ability of tannins to precipitate proteins for which the chain length seems to be the major factor. A DP > 3 is necessary for an astringent effect and the number of flavan-3-ol units is reported to be more important for an interaction with proteins than the hydroxylation pattern of the B-ring or the *cis/trans* ratio of positions 2 and 3 of ring C [39]. Furthermore, previous investigations lead to similar findings regarding the role of the OPCs’ molecular size; while a certain chain length was required for astringent effects, the capacity to precipitate proteins seems to reach a plateau for OPC above a certain DP [47].

The impact of the molecular size for the anthelmintic effect of condensed tannins has also been a topic of recent investigations [41,42,43,44,45]. Williams *et al.* observed similar effects using purified fractions from different plant sources against *Ascaris suum*. Generally, fractions with a higher mean DP (>5.4) were shown to be more effective than those consisting of smaller molecules (mDP 2.3 to 4.9), but no significant difference in the activity of the higher oligomeric fractions were observed [42], which is in accordance to our findings. On the other hand, Mohamed *et al.* observed a significant increase in the activity of OPCs from *Paeonia suffruticosa* against *C. elegans* with a molecular weight from 2100 to 4530 Da [41]. Nevertheless, investigations using purified fractions of condensed tannins [42,44,45] cannot describe the relationship between the DP and the anthelmintic as precisely as in this study using clusters of a defined DP. An increase in the activity with the molecular size up to a certain degree is typical for an unspecific tannin-like interaction of OPCs with proteins [40,46] therefore it seems likely that the active OPC clusters agglutinate certain proteins of the worms. This assumption is supported by investigations using hydrolysable tannins that revealed anthelmintic effects against different kinds of nematodes [41,48,49]. However, differences in the susceptibility among species or developmental stages within the same species [44,45,50] raise the question how “unspecific” these typical tannin-protein interactions are in nematodes.

For example, Williams *et al.* recently observed damages in the cuticle and hypodermis of *Ascaris suum* L4 larvae [42] and adult *Oesophagostomum dentatum* [45] treated with fractions or extracts from hazelnut skin rich in condensed tannins, whereas Mori *et al.* did not observe any changes in the cuticle of *C. elegans* after incubation with ellagitannins [49]. Also, none of the OPC clusters tested in our assays showed any disruptions of the cuticle of the free-living nematode *C. elegans*, which has previously shown to be a lot more resistant to external factors than the cuticle of parasitic nematodes [51].

## 3. Experimental Section

### 3.1. Plant Material and Chemicals

Leaves from *C. mucronatum* were harvested in April and May 2011 from the Bosomtwi-Atwima-Kwanwoma area in the Ashanti region of Ghana, located between 0.15–2.25°W and 5.50–7.46°N. After botanical authentication the material was air dried for two weeks at room temperature and reference samples were stored at the Institute for Pharmaceutical Biology and Phytochemistry, Muenster, Germany (voucher no. IPBP-324). If not stated otherwise, all chemicals were purchased from VWR (Darmstadt, Germany).

### 3.2. General Analytical Techniques

TLC was carried out on silica gel plates 60 (0.2 mm; Merck, Darmstadt, Germany) using ethyl acetate/water/formic acid (90:5:5 *v*/*v*/*v*) as the mobile phase. Compounds were visualized by spraying with anisaldehyde/sulphuric acid reagent followed by heating the plate to approx. 105 °C, vanillin-HCl reagent or Naturstoff reagent (diphenylboryloxyethylamin 1%).

Analytical UPLC was performed on Acquity UPLC^®^ H-class or Acquity™ Ultra Performance LC, PDA λe Detector, QDa™ Detector or Acquity™ FLR detector, autosampler, in-line degasser, Waters Empower 3^®^ Software (Waters, Milford, MA, USA).

Preparative HPLC was carried out using Quaternary Gradient Module 2545, Photodiode Array Detector 2998, Autosampler 2707, Waters Prep Degasser and Waters Fraction Collector III. Software: Waters ChromScope v1.40 Beta (Waters).

NMR spectra were recorded on an Agilent DD2 400 MHz or 600 MHz spectrometers (Agilent Technologies, Santa Clara, CA, USA). Samples were solved in chloroform-*d*_1_ or methanol-*d*_4_ and solvent peaks were set as reference at 7.260 ppm or 4.870 ppm respectively. Peracetylation of the oligomeric procyanidins was performed in pyridine/acetic acid anhydride (1:1) at room temperature for 24 h in the dark [17].

UHPLC-ESI-qTOF-MS: Separation was performed on a Dionex Ultimate 3000 RS Liquid Chromatography System (Thermo Fisher, Oberhausen, Germany) over a Dionex Acclaim RSLC 120, C18 column (2.1 × 100 mm, 2.2 µm) with a binary gradient (A: water with 0.1% formic acic; B: acetonitrile with 0.1% formic acid) at 0.8 mL/min. 0 to 9.5 min: linear from 5% to 100% B; 9.5 to 12.5 min: isocratic at 100% B; 12.5 to 12.6 min: linear from 100% to 5% B; 12.6 to 15.0 min: isocratic at 5% B. The injection volume was 2 µL. Eluted compounds were detected using a Dionex Ultimate DAD-3000 RS over a wavelength range of 200–400 nm and a Bruker Daltonics micrOTOF-QII time-of-flight mass spectrometer (Bruker, Bremen, Germany) equipped with an Apollo electrospray ionization source in negative mode at 5 Hz over a mass range of *m*/*z* 50–2000 using the following instrument settings: nebulizer gas nitrogen, 5 bar; dry gas nitrogen, 9 L/min, 220 °C; capillary voltage 3500 V; end plate offset −500 V; transfer time 100 µs, prepulse storage 10 µs; collision cell RF settings were combined to each single spectrum of 1000 summations as follows: 500 summations with 1400 Vpp + 500 summations with 350 Vpp. Internal dataset calibration (Enhanced quadratic mode) was performed for each analysis using the mass spectrum of ESI-L low concentration tunemix (Agilent Technologies) that was infused during LC reequilibration using a divert valve equipped with a 20 µL sample loop.

### 3.3. Preparation of Plant Extract and Partitions (see Figure 1)

Dried and pulverized plant material (1 kg) was defatted for 18 h by Soxhlet extraction with petroleum ether, yielding 3.6 g of extract. The remaining material (995 g) was successively extracted with ethanol water (1:1 *v*/*v*) in a drug-solvent ratio of 1:10 by Ultra-Turrax^®^ (IKA, Staufen, Germany) at 9500 rpm for 10 min under ice cooling. The suspension was centrifuged at 3000× *g* for 10 min, concentrated *in vacuo* and lyophilized. The crude extract (yield: 267 g) and all fractions obtained from the extract by the following fractionation were stored at −20 °C.

A portion of the EtOH-H_2_O extract (265 g) was partitioned between ethyl acetate by dissolving portions of 15 g of EtOH-H_2_O extract in 500 mL of water and partitioning repeatedly five times with 500 mL of EtOAc. The aqueous and organic phases were filtered (filter paper 595, S & S, Dassel, Germany) and lyophilized. Yield: 42 g of the EtOAc phase and 161 g of the aqueous phase, corresponding to 15.7% and 60.4% of the EtOH-H_2_O extract respectively.

The lyophilized EtOAc partition (8 g) was further fractionated in portions of 1 g by Fast Centrifugal Partition Chromatography (FCPC, Kromaton, Kromaton Technologies, Angers, France), mobile phase: EtOAc–heptane 9:1 (*v*/*v*), stationary phase: MeOH–H_2_O 1:9 (*v*/*v*); ascending mode, flow 8 mL/min; 1000 rpm; fraction size 16 mL). The twelve fractions (FI to FXII) obtained were investigated by TLC and fractions with similar composition were combined. 

The dried aqueous partition (10 g) was extracted three times with MeOH (400 mL), yielding 6.6 g of a MeOH soluble fraction of which 5 g were subsequently fractionated by MPLC on an RP-18 stationary phase (RP-18, 18–32 µm, 100 Å, 36 × 500 mm (BESTA Technik, Wilhelmsfeld, Germany), flow 4 mL/min, step gradient MeOH 10% (50 min)→MeOH 30% (2 h)→MeOH 50% (2 h)→MeOH (2 h), fraction size 16 mL). 

Fractions containing comparable compounds as monitored by TLC were combined to yield four subfractions H1 to H4; H1: 320–480 mL, 1.36 g; H2: 576–976 mL, 597 mg; H3: 992–1152 mL, 1.29 g; H4: 1168–1440 mL, 116 mg.

### 3.4. Isolation of Oligomeric Procyanidins with DP 1 to 3

Fractions V to XI obtained by FCPC were further subjected to preparative HPLC on an RP-18 stationary phase (Nucleodur^®^ C18 HTec, 250 × 21 mm, 5 µm, Macherey-Nagel, Düren, Germany) using a binary gradient with water (A) and acetonitrile (B) at a flow of 15.5 mL/min:

Fraction V (192 mL–256 mL; 580 mg after FCPC): *t*_0min_ 85% A 14 min isocratic, *t*_40min_ 79% A, *t*_50min_ 100% B, affording mainly compound **1** (153 mg).

Fraction VI (272 mL–384 mL; 163 mg after FCPC): *t*_0min_ 87% A 12 min isocratic, *t*_30min_ 80% A, *t*_50min_ 75% A, *t*_55min_ 100% B, yielding **1** (22 mg), **2** (0.6 mg) and **3** (3.2 mg).

Fraction VII (400 mL–544 mL; 62 mg after FCPC): *t*_0min_ 87% A 12 min isocratic, *t*_32min_ 86% A, *t*_100min_ 85% A, *t*_125min_ 67% A, *t*_130min_ 100% B, yielding **4** (0.6 mg) and **5** (1.2 mg).

Fraction VIII (560 mL–736 mL; 70 mg after FCPC), fraction IX (752 mL–816 mL; 40 mg after FCPC), fraction X (832 mL–1120 mL; 9 mg after FCPC) and fraction XI (1136 mL–1520; 20 mg after FCPC): *t*_0min_ 87% A 5 min isocratic, *t*_25min_ 86% A, *t*_55min_ 85% A, *t*_80min_ 67% A, *t*_85min_ 100% B. Compounds afforded were **4** (41.9 mg), **6** (0.38 mg) and **8** (0.32 mg).

### 3.5. Isolation of OPC with Higher DP (DP > 3)

The stationary phase obtained from separation by FCPC was further subfractionated by preparative HPLC on a diol stationary phase (Uptisphere^®^120 Å, bonding OH, 6 µm, 21.2 × 250 mm; Interchim, Montluçon, France) using a binary gradient of acetonitrile (A) and MeOH–H_2_O (95:5 *v*/*v*) (B) for isolation of OPC clusters DP3 to DP6 and one dimeric flavan-3-ol.

Gradient: *t*_0min_ 100% A, *t*_30min_ 60% A, *t*_60min_ 60% A, *t*_45min_ 100% B Flow rate: 10 mL/min.

Compounds eluted were **9** (*t*_R_ = 26 min; 9.6 mg), OPC cluster DP3 (*t*_R_ = 30 min; 103 mg), OPC cluster DP4 (*t*_R_ = 33 min; 42 mg), OPC cluster DP5 (*t*_R_ = 36 min; 23 mg) and OPC cluster DP6 (*t*_R_ = 38 min; 6 mg).

Compounds **7** (*t*_R_ = 19 min; 7.2 mg) and **8** (*t*_R_ = 20 min; 43 mg) and were afforded from OPC cluster DP3 and compound **10** (*t*_R_ = 22 min; 1.8 mg) from OPC cluster DP5 by preparative HPLC on an RP-18 stationary phase. Gradient: *t*_0min_ 95% A, *t*_3min_ 95% A, *t*_30min_ 55% A, *t*_35min_ 100% B.

Fraction H3 obtained by MPLC was subjected to subfractionation by preparative HPLC on a diol stationary phase (Uptisphere^®^120 Å, bonding OH, 6 µm, 21.2 × 250 mm; Interchim, Montluçon, France) using a binary gradient for the isolation of OPC clusters DP3 to DP10 and a polymeric fraction.

Gradient: *t*_0min_ 100% A, *t*_30min_ 60% A, *t*_40min_ 60% A, *t*_45min_ 50% A, *t*_50min_ 100% B. Flow rate: 10 mL/min.

The following subfractions were obtained: DP2 (*t*_R_ = 29 min; 8.3 mg), DP3 (*t*_R_ = 30 min; 26 mg), DP4 (*t*_R_ = 33 min; 55 mg), DP5 (*t*_R_ = 36 min; 48 mg), DP6 (*t*_R_ = 38 min; 59 mg), DP7 (*t*_R_ = 40 min; 54 mg), DP8 (*t*_R_ = 42 min; 44 mg), DP9 (*t*_R_ = 44 min; 76 mg), DP10 (*t*_R_ = 47 min; 35 mg) and polymer (56 min; 240 mg).

Compound **9** (*t*_R_ = 22 min; 7.1 mg) was isolated from fraction DP4 by preparative HPLC on an RP-18 stationary phase using a binary gradient with water (A) and acetonitrile (B) at a flow of 15 mL/min. Gradient: *t*_0min_ 90% A, *t_5_*_min_ 90% A, *t*_35min_ 60% A, *t*_45min_ 100% B.

MPLC fraction H4, mainly composed of flavonoids was further subfractionated by preparative HPLC on an RP-18 stationary phase using a binary gradient with water (A) and acetonitrile (B) at a flow of 15 mL/min for isolation of compound **13** (*t*_R_ = 18 min; 5.4 mg). Gradient: *t*_0min_ 90% A, *t*_3min_ 90% A, *t*_8min_ 86% A, *t*_27min_ 50% , *t*_32min_ 100% B.

### 3.6. Identification of Isolated Compounds

Compounds **2** to **8** were identified by UHPLC-ESI-qTOF-MS, ^1^H- and ^13^C-NMR and CD spectroscopy. Compound **1** was identified by spiking a solution of 2 mg/mL of fraction V with 2 mg/mL of epicatechin reference compound (Roth, Karlsruhe, Germany).

*Epiafzelechin-(4β→8)-epicatechin* (**2**). ESI-MS (negative mode) *m*/*z*: 561.1387 [M − H]^−^ (C_30_H_25_O_11_, calc. 561.1402); ^1^H-NMR (600 MHz, chloroform-*d*_1_), determined as epiafzelechin-(4β→8)-epicatechin-peracetate (**2a**); duplication due to dynamic rotational isomerism; two sets of signals in the ratio *ca.* 3:1): δ 7.46 (d, *J* = 8.5 Hz, 2H; H-2′/6′ (B)), 7.38 (d, *J* = 8.8 Hz, 2H; H_R_-2′/H_R_-6′ (B)), 7.10–7.08 (m, 2H; H-3′/H-5′ (B)), 7.05 (m, 2H; H_R_-3′/H_R_-5′ (B)), 7.03 (d, *J* = 1.9 Hz, 1H; H-2′ (E)), 7.03 (d, *J* = 8.3 Hz, 1H; H-5′ (E)), 6.88 (dd, *J* = 8.3, 1.8 Hz, 1H; H-6′ (E)), 6.77 (d, *J* = 2.3 Hz, 1H; H_R_-8 (A)), 6.66 (s, 1H; H-6 (D)), 6.63 (d, *J* = 2.3 Hz, 1H; H_R_-6 (A)), 6.58 (s, 1H; H_R_-6 (D)), 6.24 (d, *J* = 2.3 Hz, 1H; H-6 (A)), 5.99 (d, *J* = 2.2 Hz, 1H; H-8 (A)), 5.59 (s, 1H; H-2 (C)), 5.52 (m, 1H; H-3 (F)), 5.39 (br s, 1H; H_R_-2 (C)), 5.24 (s, 1H; H_R_-2 (F)), 5.19 (dd, *J* = 2.1, 1.3 Hz, 1H; H-3 (C)), 5.12–5.10 (m, 1H; H-3 (F)), 4.64 (d, *J* = 1.9 Hz, 1H; H_R_-4 (C)), 4.56 (s, 1H; H-2 (F)), 4.46 (d, *J* = 1.9 Hz, 1H; H-4 (C)), 3.06 (dd, *J* = 18.0, 4.9 Hz, 1H; H_R_-4b (F)), 2.98 (d, *J* = 18.2 Hz, 1H; H_R_-4a (F)), 2.93 (dd, *J* = 18.3, 5.0 Hz, 1H; H-4b (F)), 2.87 (d, *J* = 18.2 Hz, 1H; H-4a (F)). All signals correspond to literature [17].

*Epicatechin-(4β→8)-epicatechin-(4β→8)-epicatechin* (Procyanidin C1, **8**). ESI-MS (negative mode) *m*/*z*: 865.2048 [M − H]^−^ (C_45_H_37_O_18_; calc. 865.1985). ^1^H-NMR (600 MHz, chloroform-*d*_1_); determined as epicatechin-(4β→8)-epicatechin-(4β→8)-epicatechin peracetate (**6a**); duplication due to dynamic rotational isomerism: 7.29 (d, *J* = 2.2 Hz, 1H; H-2′ (H)), 7.20 (dd, *J* = 2.1, 0.7 Hz, 1H; H-6′ (H)), 7.19 (d, *J* = 1.8 Hz, 1H; H-2′ (B)), 7.17 (d, *J* = 8.4 Hz, 2H; H-2′ (E)/H-5′ (H)), 7.13 (dd, *J* = 8.5, 2.0 Hz, 2H; H-6′ (E)/H-5′ (B)), 7.09–7.06 (m, 2H; H-5′ (E)/H-6′ (B)), 6.89 (s,1H; H_R_-6 (D)), 6.76 (d, *J* = 2.3 Hz, 1H; H-8 (A)), 6.71 (s, 1H; H-6 (D)), 6.65 (d, *J* = 2.8 Hz, 1H; H-6 (A)), 6.65 (s, 1H; H-6 (G), 6.60 (s, 1H; H_R_-6 (G)), 6.26 (d, *J* = 2.3 Hz, 1H; H_R_-6 (A) or H_R_-8 (A)), 5.94 (d, *J* = 2.3 Hz, 1H; H_R_-6 (A) or H_R_-8 (A)), 5.71 (s, 1H; H_R_-2 (C)), 5.47 (m, 1H; H-3 (I)), 5.41 (s, 1H; H-2 (F)), 5.40 (m, 1H; H-3 (F)), 5.38 (s, 1H; H-2 (C)), 5.37–5.36 (m, 1H; H-3 (C)), 5.20 (s, 1H; H-2 (I)), 5.12 (s, 1H; H_R_-2 (I)), 5.11 (s, 1H; H_R_-3 (F)), 4.95 (m, 1H; H_R_-3 (C)), 4.78 (s, 1H; H-4 (C)), 4.70 (s, 1H; H-4 (F)), 4.66 (d, *J* = 4.9 Hz, 1H; H_R_-4 (F)), 4.49 (d, *J* = 2.3 Hz, 1H; H_R_-4 (C)), 3.03 (dd, *J* = 17.9, 4.7 Hz, 1H; H_R_-4b (I)), 2.96 (d, *J* = 18.5 Hz, 2H; H-4a/H-4b (I)), 2.91 (d, *J* = 18.5 Hz, 1H, H_R_-4a (I)) [18].

*Epicatechin-(4β→6)-epicatechin-(4β→8)-epicatechin* (**7**). ESI-MS (negative mode) *m*/*z*: 865.1956 [M − H]^−^ (C_4_5H_37_O_18_; calc. 865.1985). ^1^H-NMR (600 MHz, chloroform-*d*_1_); determined as epicatechin-(4β→6)-epicatechin-(4β→8)-epicatechin peracetate (**7a**): δ 7.52 (dd, *J* = 8.7, 1.8 Hz, 1H; H-6′ (B)), 7.45 (d, *J* = 1.9 Hz, 1H; H-2′ (B)), 7.28 (d, *J* = 2.3 Hz, 1H; H-2′ (E)), 7.21 (dd, *J* = 8.5, 2.1 Hz, 1H; H-6′ (E)), 7.16 d, *J* = 1.3 Hz, 1H; H-2′ (H)), 7.13 (dd, *J* = 7.8, 2.3 Hz, 1H; H-6′ (H)), 7.12 (2H; H-5′ (E)/H-5′ (H)), 7.11 (1H; H-5′ (B)), 6.85 (s, 1H; H-8 (D)), 6.66 (d, *J* = 2.2 Hz, 1H; H-8 (A)), 6.58 (d, *J* = 2.3 Hz, 1H; H-6 (A)), 6.48 (s, 1H; H-6 (G)), 5.69 (s, 1H; H-2 (C)), 5.47 (s, 1H; H-3 (I)), 5.29–5.27 (m, 1H; H-3 (F)), 5.16 (s, 1H; H-2 (I)), 4.94 (m, 1H; H-3 (C)), 4.49 (d , *J* = 1.7 Hz, 1H; H-4 (C)), 4.39 (s, 1H; H-4 (F)), 3.02 (dd, *J* = 18.1, 4.6 Hz, 1H; H-4b (I)), 2.85 (dd, *J* = 18.5, 4.9 Hz, 1H; H-4a (I)). ^13^C-NMR (150 MHz, chloroform-*d*_1_) δ154.77 (C-8a (A)), 154.22 (C-8a (D)), 151.91 (C-8a (G)), 150.30 (C-7 (A)), 149.43 (C-5 (D)), 148.83 (C-7 (D) or C-5 (G), 148.79 (C-7 (D) or C-5 (G), 147.62 C-7 (G), 142.18 (C-3′/C-4′ (H), 141.83 (C-7 (D) or C-3′ (E) or C-4′ (E)), 141.74 (C-7 (D) or C-3′ (E) or C-4′ (E)), 141.55 C-4′ (B), 137.05 (C-1′ (B)), 135.65 (C-1′ (E)), 135.42 (C-1′ (H)), 125.39 (C-6′ (B)), 124.46 (C-6′ (E)), 123.56 (C-2′ (H)), 123.23 (C-5′ (B)), 123.17 (C-5′ (E)), 122.65 (C-2′ (B)), 121.91 (H-2′ (E)), 121.72 (H-6′ (H)), 118.17 (C-8 (G)), 117.68 (C-6 (D)), 113.68 (C-4a (D)), 111.41 (C-6 (G)), 110.35 (C-4a (G)), 110.30 (C-8 (D)), 110.01 (C-4a (A)), 107.97 (C-6 (A)), 107.94 (C-8 (A)), 77.00 (C-2 (I)), 74.13 (C-2 (F)), 74.10 (C-2 (C)), 71.42 (C-3 (C)), 70.51 (C-3 (F)), 66.70 (C-3 (I)), 34.00 (C-4 (F)), 33.02 (C-4 (C)), 26.53 (C-4 (I)). Proton signals are in accordance with data published by Hör *et al* [19]. Further assignments were established by HMBC, HSQC and COSY experiments.

*Epicatechin-(4β→8)-epicatechin-(4β→6)-epicatechin* (**6**). ESI-MS (negative mode) *m*/*z*: 865.2033 [M − H]^−^ (C_45_H_37_O_18_; calc. 865.1985). ^1^H-NMR (600 MHz, chloroform-*d*_1_) determined as epicatechin-(4β*→*8)-epicatechin-(4β*→*6)-epicatechin peracetate **(8a**); duplication due to dynamic rotational isomerism: δ 7.35–6.86 (m, 9H; H-2′ (B, E, H), H-5′ (B, E, H), H-6′ (B, E, H); signals could not be assigned unequivocally), 6.78 (s, 1H; H_R_-6 (D) or H-8 (G) or H_R_-8 (G)), 6.75 (d, *J* = 2.3 Hz, 1H; H_R_-6 (A)), 6.70 (d, *J* = 2.3 Hz, 1H; H-6 (A)), 6.67 (s, 1H; H_R_-6 (D) or H-8 (G) or H_R_-8 (G)), 6.64 (s, 1H; H-6 (D) or H-8 (G) or H_R_-8 (G)), 6.31 (d, *J* = 2.3 Hz, 1H; H-6 (A)), 6.05 (d, *J* = 2.3 Hz, 1H; H_R_-8 (A)), 5.69 (s, 1H; H_R_-2 (C)), 5.24 (s, 1H; H-2 (C)), 5.18 (s, 1H; H-2 (I) or H_R_-2 (I)), 5.14 (s, 1H; H-2 (I) or H_R_-2 (I)), 4.96 (br s, 1H; H_R_-3 (C)), 4.71 (s, 1H; H-4 (C)), 4.47 (d, *J* = 2.1 Hz, 1H; H_R_-4 (C)), 4.08 (s, 1H; H-4 (F)), 2.95–2.81 (m, 4H; H4a/H-4b and H_R_-4a/H_R_-4b). Signals for H-2 (F), H_R_-2 (F), H-3 (F), H_R_-3 (F), H-3 (I) and H_R_-3 (I) could not be assigned, but all signals are in accordance with literature [19].

*Epicatechin-(6′→8)-epicatechin* (**5**). ESI-MS (negative mode) *m*/*z*: 577.1392 [M − H]^−^ (C_30_H_25_O_12_; calc. 577.1351). ^1^H-NMR (600 MHz, methanol-*d*_4_) δ 7.23 (s, 1H; H-2 (B)), 6.79 (d, *J* = 2.0 Hz, 1H; H-2 (E)), 6.73 (s, 1H; H-5 (B)), 6.66 (d, *J* = 8.2 Hz, 1H; H-5 (E)), 6.61 (dd, *J* = 8.2, 2.0 Hz, 1H; H-6 (E)), 6.10 (s, 1H; H-6 (D)), 5.89 (d, *J* = 2.3 Hz, 1H; H-6 (A)), 5.82 (d, *J* = 2.3 Hz, 1H; H-8 (A)), 4.78 (s, 1H; H-2 (F)), 4.70 (s, 1H; H-2 (C)), 4.20 (m, *J* = 4.9, 1.7 Hz, 1H; H-3 (F)), 4.04–4.02 (m, 1H; H-3 (C)), 2.90 (dd, *J* = 16.7, 4.7 Hz, 1H; H-4b (F)), 2.73 (dd, *J* = 16.7, 3.8 Hz, 1H; H-4a (F)), 2.66 (d, *J* = 16.6 Hz, 1H; H-4b (C)), 2.44 (dd, *J* = 16.8, 4.8 Hz, 1H; H-4a (C)). ^13^C-NMR (150 MHz, methanol-*d*_4_) δ 157.88 (C-5 (A) or C-7 (A)), 157.77 (C-5 (A) or C-7 (A)), 157.47 (C-8a (A)), 157.06 (C-5 (D)), 155.00 (C-7 (D)), 153.68 (C-8a (D)), 145.61, 145.47, 145.32, 145.06 (C-3 (B) or C-4 (B) or C-3 (E) or C-4 (E), 131.96 (C-1 (E)), 131.82 (C-1 (B)), 125.22 (C-6 (B)), 119.93 (C-5 (B)), 119.63 (C-6 (E)), 116.01 (C-5 (E)), 115.78 (C-2 (B)), 115.04 (C-2 (E)), 108.15 (C-8 (D)), 100.53 (C-4a (F)), 99.78 (C-4a (C)), 96.25 (C-6 (D)), 96.12 (C-8 (C)), 96.01 (C-6 (C)), 79.49 (C-2 (F)), 78.09 (C-2 (C)), 66.84 (C-3 (F)), 65.85 (C-3 (C)), 29.35 (C-4 (C)), 28.95 (C-4 (F)).

All signals were in accordance with literature [30], the assignment of the protons at position 4 of the C-ring and of the F-ring was revised according to correlations obtained from NOE and HMBC spectra.

*Epicatechin-(4β→8)-epicatechin-(4β→8)-epicatechin-(4β→8)-epicatechin* (cinnamtannin A2, **9**). ESI-MS (negative mode) *m*/*z*: 1153.2701 [M − H]^−^ (C_60_H_50_O_24_; calc. 1153.2619). ^1^H-NMR (400 MHz, chloroform-*d*_1_) determined as epicatechin-(4β→8)-epicatechin-(4β→8)-epicatechin-(4β→8)-epi-catechin peracetate (**9a**); duplication due to dynamic rotational isomerism. Numbers in brackets indicate the sequnce of epicatechin units starting from A.: δ 7.38–6.94 (m, H-2′/H-5′/H-6′ of units A/B/C/D), 6.89 (s, 1H; H_R_-6 (B)), 6.76 (d, *J* = 2.3 Hz, 1H; H-8 (A)), 6.74 (s, 1H; H-6 (B)), 6.70 (s, 1H; H-6 (C)), 6.65 (s, 1H; H-6 (A)), 6.64 (s, 1H; H-6 (D)), 6.61 (s, 1H; H_R_-4 (C)), 6.58 (s, 1H; H_R_-6 (D)), 6.25 (d, *J* = 2.3 Hz, 1H; H_R_-6 (A)), 5.88 (d, *J* = 2.5 Hz, 1H; H_R_-8 (A)), 5.73 (s, 1H; H_R_-2 (A)), 5.47 (br s, 2H; H-3(D)/H_R_-3 (D)), 5.44 (s, 1H; H-2 (A)), 5.35 (s, 1H; H-3 (B) or H_R_-2 (C)), 5.33 (s, 1H; H-3 (C)), 5.31 (s, 1H; H-3 (A)), 5.28 (s, 1H; H-2 (C) or H_R_-3 (C)), 5.20 (s, 1H; H-2 (D) or H_R_-2 (D)), 5.19 (s, 1H; H-2 (D) or H_R_-2 (D)), 5.15 (s, 1H; H_R_-3 (B)), 4.95 (s, 1H; H_R_-3 (A)), 4.84 (s, 1H; H-4 (B)), 4.79 (s, 1H; H_R_-4 (B)), 4.76 (s, 1H; H-4 (A)), 4.66 (s, 1H; H-4 (C)), 4.61 (s, 1H; H_R_-6 (C)), 4.56 (s, 1H; H_R_-2 (B)), 4.51 (d, *J* = 2.7 Hz, 1H; H_R_-4 (A)), 3.02 (m, 4H; H-4a/H-4b and H_R_-4a/H_R_-4b (D)), 2.38–1.24 (m, acetates) [25]. ^13^C-NMR (150 MHz, chloroform-*d*_1_) δ 155.04 (C-8a (A), 151.97 (C-8a (B)), 151.88 (C-8a (C)), 151.79 (C-8a (D)), 150.01 (C-7 (A)), 149.85 (C-5 (A)), 148.86 (C-5 (C)), 148.75 (C-5 (B)), 148.66 (C-5 (D)), 147.59 (C-7 (B)), 147.49 (C-7 (C)), 147.41 (C-7 (D)), 142.19 (C-3′(A) or C-4′(A), 142.11 (C-3′(A) or C-4′(A) or C-4′ (B) or C-3′ (C) or C-4′ (D)), 142.02 (C-3′ (B), 141.94 C-4′ (C), 136.63 (C-1′(A), 135.88 (C-1′ (D), 135.64 (C-1′ (B), 135.51 (C-1′ (C), 124.93 (C-6′ (A), 124.49 (C-6′ (B), 124.11 (C-6′ (D)), 123.69 (C-6′ (C), 123.29 (C-5′ (D)), 123.26 (C-5′ (C)), 122.92 (C-5′ (A)), 122.91 (C-2′)), 122.01 C-5′ (B)), 121.88 (C-6′ (2)), 121.57 (C-2′ (B) or C-2′ (C), 118.33 (C-8 (C)), 117.95 (C-8 (B)), 117.54 (C-8 (D)), 112.71 (C-4a (C)), 112.43 (C-4a (B)), 111.79 (C-4a (A)), 111.47 (C-6 (B)), 110.89 (C-6 (D)), 110.81 (C-6 (C)), 109.91 (C-4A (D)), 109.50 (C-6 (A)), 108.35 (C-8 (A)), 76.98 (C-2 (D)), 75.48 (C-2 (B)), 75.24 (C-2 (C)), 74.83 (C-2 (A)), 71.64 (C-3 (C)), 71.44 (C-3 (B)), 70.85 (C-3 (A)), 66.70 (C-3 (D)), 35.41 (C-4 (B)), 35.34 (C-4 (C)), 34.42 (C-4 (A)), 26.51 (C-4 (D)). Proton signals are in accordance with Hör *et al.* [19]. Assignments for the carbon atoms were established by HMBC, HSQC and COSY experiments.

*Pentameric Procyanidin* **10**. ESI-MS (negative) *m*/*z*: 1441.3264 [M − H]^−^ (C_60_H_61_O_30_; calc.1441.3326). ^1^H-NMR (600 MHz, chloroform-*d*_1_) δ 7.55–6.95 (m, 15H), 6.76 (s, 1H; H-6 (B) or H-6 (C) or H-6 (D)), 6.75 (s, 1H; H-6 (B) or H-6 (C) or H-6 (D)), 6.73 (s, 1H; H-6 (B) or H-6 (C) or H-6 (D)), 6.65 (s, 1H, H-6 (E)), 6.64 (s, 1H; H-6 (A)), 6.27 (d, *J* = 2.2 Hz, 1H; H-8 (A)), 5.75 (s, 1H; H-2 (A)), 5.49 (br s, 1H; H-3 (E)), 5.43 (s, 1H; H-2 (C)), 5.40 (s, 2H; H-3 (D)), 5.35 (br s, 1H; H-3 (B)), 5.32 (s, 1H; H-2 B)), 5.30 (s, 1H; H-3 (C)), 5.20 (s, 1H; H-2 (E)), 5.10 (s, 1H; H-2 (D)), 4.97 (s, 1H; H-3 (A)), 4.82 (s, 1H; H-4 (D)), 4.79 (s, 1H), 4.78 (d, *J* = 4.7 Hz, 1H; H-4 (C)), 4.67 (s, 1H; H-4 B)), 4.52 (d, *J* = 2.2 Hz, 1H; H-4 (A)), 3.09 (dd, *J* = 18.1, 4.3 Hz, 1H; H-4b), 2.98 (d, *J* = 7.5 Hz, 1H; H-4a). ^13^C-NMR (150 MHz, chloroform-*d*_1_) δ 151.64 (C-8a (D) or C-8a (E)), 151.57 (C-8a (C) or C-8a (D)), 151.53 (C-8a (B) or C-8a (C)), 149.72 (C-7 (A)), 148.33 (C-5 (A)), 148.22 (C-5 (B)), 147.28 (C-5 (D)), 147.22 (C-5 (C)), 147.07 (C-5 (D)), 142.22, 142.00, 141.81, 141.78, 141.67, 141.51, 141.45, 141.31, 141.18, 140.73 (10C; C-3′/C-4′ (A–E)), 135.62 (C-1′ (E)), 117.73 (C-8 (B)), 117.47 (C-8 (D)), 117.22 (C-8 (E)), 112.22 (C-4a (A)), 112.14 (C-4a (B)), 111.44 (C-4a C)), 110.67 (C-6 (E)), 110.50 (C-6 (A)), 109.68 (C-4a (E)), 107.59 (C-8 (A)), 74.87 (C-2 B)), 74.58 (C-2 (C)), 73.86 (C-2 (A)), 71.39 C-3 (B)), 71.36 C-3 (D)), 70.83 (C-3 (A)), 70.45 (C-3 (C)), 66.43 (C-3 (E)), 35.06 (C-4 (D)), 34.99 (C-4 (B)), 34.17 (C-4 (C)), 32.74 (C-4A)), 26.05 (C-4 (E)).

*Isovitexin* (**11**). ESI-MS (negative mode) *m*/*z*: 431.1027 [M − H]^−^ (C_21_H_19_O_10_; calc. 431.0984). ^1^H-NMR (600 MHz, methanol-*d*_4_) δ 7.87 (dd, *J* = 8.9/2.0 Hz; 2H; H-2′/H-5′), δ 6.95 (dd, *J* = 8.9/2.0; 2H; H-3′/H-5′), 6.64 (s, 1H; H-3), 6.54 (s, 1H; H-8), 4.19–3.47 (6H, glucose). All signals correspond to literature [52].

*Isoorientin* (**13**). ESI-MS (negative mode) *m*/*z*: 447.0971 [M − H]^−^ (C_21_H_19_O_11_; calc. 447.0933). ^1^H-NMR (600 MHz, methanol-*d*_4_) δ 7.39 (d, *J* = 8.2 Hz, 1H; H-5), δ 7.38 (s, 1H; H-3), 6.91 (d, *J* = 8.2 Hz, 1H; H-6), 6.56 (s, 1H; H-8), 6.50 (s, 1H; H-2′), 4.90 (d, *J* = 9.9 Hz, 1H; H-1′′), 4.20–4.14 (m, 1H; H-4′′), 3.88 (dd, *J* = 12.1, 2.3 Hz, 1H; H-6′′a), 3.74 (dd, *J* = 12.1, 5.4 Hz, 1H; H-6′′b), 3.49–3.47 (m, 2H; H-2′′/H-3′′), 3.45–3.40 (m, 1H; H-5′′). ^13^C-NMR (150 MHz, methanol-*d*_4_) δ 184.05 (C-4), 166.32 (C-7), 162.09 (C-5), 158.80 (C-9), 151.18 (C-4′), 147.14 (C-3′), 123.59 (C-1′), 116.84 (C-6′), 114.16 (C-2′), 109.30 (C-6), 105.16 (C-10), 103.94 (C-3), 95.28 (C-8), 82.68 (C-5′′), 80.20 (C-3′′), 75.36 (C-1′′), 72.63 (C-4′′), 71.83 (C-2′′), 62.92 (C-6′′). Data were compared to literature [53,54] using DMSO as a solvent, but for **13** dissolved in MeOD, assignments had to be newly established.

### 3.7. Tannin-Depleted Extract: Precipitation of Proanthocyanidins with PVPP 

7.5 g of Polyclar AT (Serva, Heidelberg, Germany) were added to 1.5 g of the ethyl acetate partition solution, dissolved in 200 mL H_2_O and kept at room temperature for 35 min under constant shaking (135 cycles/min; GFL reciprocating shaker 3018, GFL, Burgwedel, Germany). The suspension was then centrifuged at 2500× *g* (Laborfuge 400e, Heraeus, Hanau, Germany) for 10 min and the supernatant was lyophilized for further investigation (yield: 173 mg). Proanthocyanidin depletion was proven by TLC using vanillin-hydrochloric acid reagent for specific PAC detection as well as by UPLC.

### 3.8. Identification of Flavonoids by HPLC

A solution of the EtOAc partition (0.5 mg/mL) was spiked with 200 µL of solutions of vitexin, isovitexin, isoorientin, isoquercitrin and hyperosid (0.5 mg/mL) (Roth) respectively. The samples were subjected to analytical UPLC (Waters ACQUITY^®^) on an RP-18 stationary phase (HSS T3, 1.8 µm, 2.1 × 100 mm) using a binary gradient of 0.1% formic acid (A) and acetonitrile (B): *t*_0min_ 90% A, *t*_8min_ 86% A, *t*_10min_ 100% B. Flow rate: 0.5 mL/min. By UV detection at λ = 340 nm and mass detection (negative mode), four flavonoids were identified: vitexin (**12**; *t*_R_ = 8.51 min), isovitexin (**11**; *t*_R_ = 8.92 min), isoorientin (**13**; *t*_R_ = 6.46 min) and isoquercitrin (**14**; *t*_R_ = 8.96 min). 

### 3.9. Anthelmintic Assay

#### 3.9.1. Monoxenic and Axenic Maintenance of *Caenorhabditis Elegans*

Cultures of wildtype *C. elegans* (N2 Bristol strain) were maintained as described by [12]. Monoxenic cultures were grown at 20 °C on petri dishes containing Nematode Growth Medium supplemented with 800 µL of *Escherichia coli* OP50 strain as a food source [55].

Age synchronous cultures were obtained by the alkaline bleaching method described by Ndjonka *et al.* [56]: Worms were rinsed from the Petri dish with M9 buffer solution (3 g KH_2_PO_4_, 6 g Na_2_HPO_4_, 5 g NaCl, 0.25 g MgSO_4_ 7H_2_O in 1 L of water [57]), centrifuged at 2000× *g* for 1 min and treated with alkaline solution (600 µL sodium hypochlorite solution, (Sigma-Aldrich, Steinheim, Germany), 100 µL 10 M sodium hydroxide solution, 1.300 mL H_2_O) for 7 min after the supernatant was removed. 

For the *in vitro* assay, synchronous cultures of *C. elegans* were initiated in axenic liquid medium composed of 3.0 g Bacto™ yeast extract (Becton-Dickinson, Heidelberg, Germany), 3.0 g soy peptone from casein (Sigma-Aldrich), 1.0 g dextrose, and 0.25 mL cholesterol solution (5 mg/1.0 mL in ethanol) in 100 mL of H_2_O and autoclaved. To start the axenic culture, synchronous worm cultures were seeded into the axenic medium supplemented with 0.05% hemoglobin (stock solution: 5% (*w*/*v*) of bovine hemoglobin (Sigma-Aldrich) in 0.1 M KOH, autoclaved for 10 min) [58] and 0.1 % (*v*/*v*) penicillin/streptomycin solution (10,000 U/10,000 µg/mL; Gibco/Invitrogen, Darmstadt, Germany) [56]. 

#### 3.9.2. *In Vitro* Screening

*In vitro* testing was performed as described by [12]. Briefly, stock solutions of the EtOH-H_2_O extract, the respective fractions (50 mg/mL) and purified compounds (5 mM) were prepared with M9 buffer solution using dimethyl sulfoxide (DMSO) as a solubilizer and centrifuged at 2000× *g* for 1 min. The final concentration of DMSO did not exceed 1% (*v*/*v*). Aliquots of the stock solution were added to a 24-well microtiter plate containing culture medium (50 mL 20 % (*w*/*v*) dextrose solution, 500 µL of a solution from cholesterol 5 mg/mL in ethanol, 500 µL 1 M CaCl_2_, 500 µL 1 M MgSO_4_, 12.5 mL 1 M KH_2_PO_4_/K_2_HPO_4_ and 500 µL penicillin/streptomycin solution (10,000 U/10,000 µg/mL) in 500 mL M9 buffer solution) to a final volume of 500 µL per well. Test concentrations ranged from 0.05 to 5 mg/mL for fractions and from 1 to 1000 µM for purified compounds.

Each substance was tested in 4 replicates per treatment and each experiment was independently performed in triplicate. A solution of levamisole hydrochloride (40 mM) (AppliChem, Darmstadt, Germany) served as a positive control; DMSO 1% (*v*/*v*) was used as a negative control.

10 to 20 worms (L4 larvae or young adults) were incubated with the respective test substance at 20 °C and the mortality was assessed after 72 h by counting the number of dead worms under a dissecting microscope: worms that were immotile and completely straight were counted as dead if they did not respond when prodded with an eyelash. The percentage of dead worms was calculated as the number of dead worms in relation to the total number of worms per well.

### 3.10. Statistical Analysis

Data obtained from the *in vitro* assay were analyzed using GraphPad Prism^®^ Ver. 3 (GraphPad Software, Inc., La Jolla, CA, USA). Mean values of mortality rates were compared by a one-way ANOVA test followed by a Tukey’s Test for multiple comparison. A *p*-value < 0.05 compared to the negative control was considered to be significant. 

## 4. Conclusions

Unsubstituted oligomeric procyanidin units were found to be the active components of a hydroethanolic leaf extract of *C. mucronatum*, a plant which is traditionally used in West Africa as an anthelmintic remedy. Structure elucidation of the isolated OPCs revealed that they are almost entirely composed of epicatechin units with 4β→8 and 4β→6 linkages. The activity of these compounds increased with their molecular size showing a maximum activity from DP4 to DP10. These findings point towards an interaction of OPCs with so far unidentified proteins of the target organism. Our findings confirm and rationalize the traditional use of *C. mucronatum* and provide further insight into the anthelmintic activities of condensed tannins. Further studies evaluating the potential of extract and isolated clusters against different parasitic nematodes would be desirable. Additionally, the precise mode of action of condensed tannins apart from few microscopic observations remains to be investigated.

## Abbreviations

CD: circular dichroism; DP: degree of polymerization; EC: epicatechin; EtOH: ethanol; EtOAc: ethyl acetate; FCPC: fast centrifugal partition chromatography; HPLC: high pressure liquid chromatography; MeOH: methanol; NC: negative control; OPC: oligomeric procyanidins; PC: procyanidins; RT: room temperature; STH: soil-transmitted helminths; TLC: thin layer chromatography; UHPLC: ultra-high pressure liquid chromatography.
